# Acrylamide in Cooked Sprouts of Mung Bean (*Vigna radiata*)

**DOI:** 10.14252/foodsafetyfscj.D-23-00001

**Published:** 2023-04-08

**Authors:** Kazuhiro Chiku, Ai Yamada, Yui Shibasaki, Yoshiki Makino, Taidoh Komatsuzaki, Mitsuru Yoshida

**Affiliations:** Department of Food Science and Technology, Faculty of Applied Life Science, Nippon Veterinary and Life Science University, 1-7-1 Kyonancho, Musashino, Tokyo 180-8602, Japan

**Keywords:** exposure estimation, pan-fry, parch, risk assessment, stir-fry, vegetable.

## Abstract

We investigated the time-dependent acrylamide formation in mung bean sprouts during stir-frying under high and medium heat conditions. The acrylamide concentration range detected using the 3-mercaptobenzoic acid derivatization LC-MS/MS method was from below 29 ng/g [limit of detection (LOD)] to 6,900 ng/g. We also investigated the acrylamide levels in mung bean sprouts cooked using four methods while retaining their fresh firm texture using the thiosalicyclic acid derivatization LC-MS/MS method. The acrylamide concentration in microwave oven-cooked sprouts was below 16 ng/g (LOD). The samples cooked by stir-frying, parching, or boiling contained an acrylamide concentration above the LOD but below 42 ng/g [limit of quantification (LOQ)], except for one replicate of a stir-fried sample, whose acrylamide concentration was 42 ng/g. Bean sprouts are popular affordable vegetables, and when stir-fried, their acrylamide concentration is assumed to strongly affect the exposure of the Japanese population to acrylamide. Because the acrylamide concentration range of fried bean sprouts is as broad as mentioned above, the selection of a representative concentration value is difficult. A precise survey and data about acrylamide formation in relation to the bean sprout components before heating, their changes occurring during storage, and the cooking methods and conditions used are needed to estimate the exposure of the Japanese to acrylamide. Here, we showed that rinsing the sprouts before frying and frying them for a short time while mixing them well, while retaining the fresh firm texture to avoid burning and shriveling the sprouts is effective in decreasing the amount of acrylamide formed.

## 1. Introduction

Acrylamide, which is genotoxic and carcinogenic, has been reported to form in high-temperature processed or cooked foods for the first time in 2002^1^^,^^2^^)^. Subsequently, studys assessing the acrylamide content in processed or cooked foods have been conducted worldwide. Their results have been analyzed together with food intake data to estimate the exposure to acrylamide and its corresponding risk. The European Food Safety Authority (EFSA)^3^^)^ estimated that the chronic dietary exposure of children to acrylamide is 0.5–1.9 µg/kg body weight (bw)/day, on average, and that of adolescents, adults, the elderly, and the very elderly was estimated to be 0.4–0.9 µg/kg bw/day, on average, depending on the survey and age group analyzed. In the EFSA estimation, the main contributors to the total acrylamide exposure in toddlers, other children, and adolescents were fried potato products (except for potato chips, i.e. potato crisps in Europe, and snacks), representing up to half of the total exposure, followed by soft bread, breakfast cereals, biscuits, crackers, crispbread, and other products made of cereals and potatoes. Coffee is another main contributor to acrylamide exposure in adults, the elderly, and the very elderly. The U.S. Food and Drug Administration (FDA)^4^^)^ estimated that the mean dietary intake for 2-year-olds and older people was 0.36 µg/kg bw/day based on the data from surveys of 2011–2015 . They reported that French fries and potato products, breakfast cereals, cookies, potato chips, and crackers continued to be significant contributors to acrylamide exposure, and infant snack foods were identified as more important contributors than jarred infant foods. The Food Standards Australia New Zealand (FSANZ)^5^^)^, which reported that the lower and upper bound acrylamide exposure of consumers is 1–2 and 2–4 µg/kg bw/day, respectively, reported that vegetables and pulses are some of the main sources of dietary acrylamide exposure across the age groups assessed. The Government of Hong Kong^6^^)^ and Zhu et al of the Chinese Center for Disease Control and Prevention^7^^)^ have estimated that the average dietary exposure to acrylamide is 0.21 and 0.175 µg/kg bw/day, respectively, and have pointed out that vegetables are the main source of acrylamide exposure, accounting to more than 50% of the total exposure.

In 2016, the Food Safety Commission of Japan (FSCJ)^8^^,^^9^^)^ performed risk assessment on heat-generated acrylamide in foods. The acrylamide oral intake was estimated to be 0.240 µg/kg bw/day, 56.0% of which was derived from fried/sautéed vegetables. Fried/sautéed bean sprouts were the food that contributed the most to the total acrylamide exposure, reaching 28% of the total exposure.

Nonetheless, the data on the acrylamide content of stir-fried (pan-fried, sautéed) bean sprouts are limited. Bean sprouts are often stir-fried with other vegetables and meat, and estimating the acrylamide derived solely from the bean sprouts is difficult when analyzing a fried vegetable mixture. In 2014, the minimum, maximum, mean, and median acrylamide contents in fried bean sprouts were reported to be 0.028 mg/kg (28 ng/g), 0.22 mg/kg (220 ng/g), 0.087 mg/kg (87 ng/g), and 0.078 mg/kg (78 ng/g), respectively, by the Ministry of Agriculture, Forestry and Fisheries (MAFF) of Japan^10^^)^. In 2015, additional data on the acrylamide content of vegetables cooked at high temperatures were reported as a result of a project by MAFF^11^^)^. For the acrylamide exposure estimation in the risk assessment performed by FSCJ in 2016 described above, a fried/sautéed bean sprout acrylamide concentration of 752 ng/g, which was obtained as the average of three replicates of bean sprouts stir-fried for 2 and 7 min in the project, was adopted as the concentration of fried/sautéed bean sprouts^8^^,^^9^^)^. Conversely, a concentration value of 19 µg/kg (ng/g), which is the mean of the 1–35 µg/kg (ng/g) acrylamide concentration range of stir-fried mung bean sprouts, was adopted for the exposure assessment performed in Hong Kong^6^^)^; however, the corresponding cooking conditions were not reported. In addition, Ishihara et al^12^^)^ reported an average acrylamide concentration of 2,210 ppb (ng/g) in 100 g of bean sprouts parched (dry-roasted, stir-fried without oil) for 4 min using a Teflon-lined frying pan with a diameter of 26 cm under strong heat. Noda et al^13^^)^ reported acrylamide concentrations of approximately 100 µg/100 g (1,000 ng/g) of raw sample prior to heating and of about 200 µg/100 g (2,000 ng/g) of raw sample prior to heating after parching 100 g of mung bean sprouts using a Teflon-lined frying pan with a diameter of 24 cm on strong heat for 3 min and 9 min, respectively. As previously reported, the acrylamide level range of stir-fried bean sprouts is very broad, being comparable to that of fried potato-based products. This broad range is caused by differences in the asparagine and reducing sugar contents of the raw sprouts, the cooking temperature used, the cooking time, the mixing method and frequency, and the shape and the type of material of the pan.

Since bean sprouts are popular affordable vegetables that are supplied throughout the year in Japan, their intake is rather large (0.088 g/kg bw/day)^8^^,^^9^^)^. In addition, they are often consumed as components of stir-fried dishes. Therefore, the value adopted as the representative concentration of acrylamide in stir-fried bean sprouts strongly affects the acrylamide exposure estimation, which is one of the major factors in risk assessment together with hazard characterization (toxicity evaluation). FSCJ^8^^,^^9^^)^ reported a total acrylamide oral intake of 0.240 µg/kg bw/day when 752 ng/g was adopted as the acrylamide concentration value of fried/sautéed bean sprouts. They also reported that the total acrylamide oral intake was 0.154 and 0.158 µg/kg bw/day when the value of 95 ng/g was adopted as the representative value of the acrylamide concentration of fried/sautéed bean sprouts using the Monte Carlo simulation and point estimate method, respectively.

Bean sprouts are sometimes stir-fried for a long time until their texture becomes soft; however, they are also often cooked for a short time in orider to maintain a fresh firm texture. For the risk assessment, FSCJ adopted the acrylamide concentration value obtained from well-fried softened bean sprouts, thus avoiding an underestimation of the exposure to acrylamide.

Here, we investigated the time-dependent acrylamide formation in mung bean sprouts during stir-frying under high and medium heat to determine the variation in the concentration of acrylamide formed in the sprouts. We also investigated the acrylamide levels in mung bean sprouts cooked using four methods, while maintaining their fresh firm texture. Based on these results, an appropriate representative value of the acrylamide concentration of stir-fried bean sprouts for exposure estimation in risk assessment is discussed. We also provide useful information for reducing the intake of acrylamide from bean sprouts.

## 2. Materials and Methods

### 2.1 Materials

Mung bean sprouts were purchased from supermarkets in Musashino City, Tokyo, Japan and cooked on the day of purchase. Acrylamide-^13^C_3_ (+ 100 ppm hydroquinone) was purchased from Cambridge Isotope Laboratories, Inc. (Tewksbury, MA, USA). The acrylamide standard, thiosalicyclic acid, activated charcoal (small fragments, 0.2–1.0 mm), methanol, ethyl acetate, hydrochloric acid, sodium hydroxide, and dimethyl sulfoxide were purchased from FUJIFILM Wako Pure Chemical Corporation (Osaka, Japan). 3-Mercaptobenzoic acid (3-MBA) was purchased from Tokyo Chemical Industry Co., Ltd. (Tokyo, Japan). Lead (II) acetate trihydrate was purchased from Kanto Chemical Co., Inc. (Tokyo, Japan). Magnesium sulfate was purchased from Sigma-Aldrich Japan (Tokyo, Japan).

### 2.2 Cooking the Mung Bean Sprouts

#### 2.2.1 Stir-frying the mung bean sprouts

One teaspoon of canola oil (J-Oil Mills, INC., Tokyo, Japan) was spread on a Teflon-coated pan (WP-6294, with a top and bottom diameter, and a depth of 28, 20, and 8 cm, respectively; Wahei Freiz MS Corporation, Niigata, Japan) and placed on an induction heating (IH) heater (KZ-PG33, Panasonic Corporation, Tokyo, Japan) at 700 or 1,400 W for 1 min. Then, 200 g of mung bean sprouts were added to the pan and heated. They were mixed once per second using a beater at 700 and 1,400 W for 5–20 and 4–13 min, respectively. The mung bean sprouts whose radicles had not been removed and which were rinsed with running water in a bowl for 1 min or not were used in the stir-fry experiment. Three replicates were performed by three different people for each cooking condition tested. The fried samples were frozen at −20°C until derivatization for LC-MS/MS analysis.

#### 2.2.2 Cooking the mung bean sprouts while retaining their fresh firm texture

The mung bean sprouts (200 g) with radicles were rinsed using running water in a bowl for 15 s. They were cooked using the following four methods (2.2.2.1–2.2.2.4) to retain their fresh firm texture. The moisture loss during cooking was estimated by measuring the weight loss after cooking. Each cooking method was repeated three times. The fried samples were frozen at −20°C until derivatization for LC-MS/MS analysis.

##### 2.2.2.1 Stir-fry method

The mung bean sprouts were cooked for 5 min as described in section 2.2.1. The IH heater was operated at 700 W.

##### 2.2.2.2 Parch method

The mung bean sprouts were cooked as described in 2.2.2.1, except that canola oil was not used.

##### 2.2.2.3 Boil method

Mung bean sprouts (200 g) were added to 2 L of boiling water in a stainless-steel pan (a top and bottom diameter, and a depth of 26, 22, and 11 cm, respectively) and boiled for 1.5 min by operating the IH heater at 700 W.

##### 2.2.2.4 Microwave oven cooking method

Mung bean sprouts (200 g) were placed in a polypropylene container with a lid (No. 4973430406758, 17 × 15 × 8 cm^3^; Daiso Industries Co., Ltd., Hiroshima, Japan) and heated for 2 min using a microwave oven (RE-SW10; Sharp Corporation, Osaka, Japan) at 500 W.

### 2.3 3-MBA Derivatization of Acrylamide

3-MBA derivatization was carried out as described by Jezussek^14^ to analyze the acrylamide content of stir-fried mung bean sprouts in a time-course heating study. A sample of stir-fried mung bean sprouts (5 g) was mixed with 10 µL of 500 µg/mL acrylamide-^13^C_3_ and 35 mL of ultrapure water. The sample was homogenized for 2 min using a homogenizer (HG-200; Hisiang Tai, New Taipei, Taiwan) and centrifuged at 10,000 × *g* for 5 min. The supernatant was filtered using a No. 5A filter paper (Toyo Roshi Kaisha Ltd., Tokyo, Japan), and 20 mL of the filtrate was collected. A column was prepared by packing 0.4 g of defatted cotton at the bottom of a 25 mL plastic syringe, followed by 0.4 g of activated charcoal, and 0.15 g of defatted cotton at the top. Then, it was conditioned by passing 5 mL of methanol through it, followed by 20 mL of ultrapure water. The filtrate was loaded onto the column and the absorbed acrylamide was eluted with 5 mL of methanol after sufficiently dehydrating the column through aeration. The methanol was evaporated at 30°C for 1 h using a centrifugal evaporator (CVE-2100, EYELA, Tokyo, Japan). The concentrated sample was transferred to a 5 mL sample tube, and 10 µL of dimethyl sulfoxide was added to it. After freezing the sample at –80°C, it was concentrated to less than 1 mL by freeze-drying it for approximately 3 h. A volume of 100 µL of 48 mg/mL 3-MBA in 1 mol/L sodium hydroxide was added to the concentrated sample, and the mixture was incubated for 3 h at 4°C in the dark. The residual 3-MBA was precipitated by adding 100 µL of a saturated lead (II) acetate aqueous solution and separated via centrifugation. Three drops of 5 mol/L hydrochloric acid were added to the collected supernatant, and the precipitated lead chloride was removed via centrifugation. The 3-MBA derivative of acrylamide was extracted thrice from the supernatant using ethyl acetate (0.5 mL) and dehydrated using magnesium sulfate. After centrifugation, the supernatant was collected, and the solvent was completely removed using a centrifugal evaporator. The sample was dissolved in 100 µL of methanol and filtered using a 0.45 µm filter (Minisart Syringe Filter, Sartorius, Göttingen, Germany) before the LC-MS/MS analysis.

### 2.4 Thiosalicyclic Acid Derivatization of Acrylamide

We used thiosalicyclic acid, which is less expensive and more easily available than 3-MBA, for derivatization referring the method described by Jezussek^14^^)^ to analyze the acrylamide content of mung bean sprouts cooked while retaining their fresh firm texture. A mass of 5 g of a stir-fried sprout sample was mixed with 10 µL of 100 µg/mL acrylamide-^13^C_3_ and 35 mL of ultrapure water. The samples were homogenized and centrifuged at 10,000 × *g* for 10 min. The supernatant was then filtered. For the stir-fried sprouts, 30 mL of the supernatant was mixed with 30 mL of n-hexane and shaken vigorously twice to remove the canola oil. A column was prepared by packing 0.4 g of defatted cotton at the bottom of a 25 mL plastic syringe, followed by 0.5 g of activated charcoal and 0.2 g of defatted cotton at the top. Then, it was conditioned by adding at least 5 mL of methanol to it, followed by at least 20 mL of ultrapure water. The sample solution (20 mL) was loaded onto the column and the absorbed acrylamide was eluted with 5 mL of methanol after thoroughly dehydrating the column via aeration and suction for 10 min using a vacuum pump (Yamato Scientific Co., Ltd., Tokyo, Japan). The eluate was concentrated to approximately 1 mL by performing centrifugal evaporation for 50 min. A volume of 100 µL of 48 mg/mL thiosalicyclic acid in 1 mol/L sodium hydroxide was added to the concentrated samples, and these were incubated for 3 h at 4°C in the dark. The residual thiosalicyclic acid was precipitated by adding 100 µL of a saturated lead (II) acetate aqueous solution and separated by centrifugation at 10,000 × *g* for 5 min. Approximately 6 drops of 5 mol/L hydrochloric acid were added to the collected supernatant, and the precipitated lead chloride was removed via centrifugation. The thiosalicyclic acid derivative of acrylamide was extracted twice from the supernatant using ethyl acetate (0.5 mL) and dehydrated using magnesium sulfate. After centrifugation, the supernatant was collected, and the solvent was completely removed using a centrifugal evaporator. The sample was dissolved in 100 µL of methanol and filtered using a 0.45 µm filter before LC-MS/MS analysis.

### 2.5 LC-MS/MS Analysis

The LC-MS/MS analysis of the acrylamide derivatives was carried out under the following conditions. We used the HPLC Prominence System (Shimadzu Corporation, Kyoto, Japan); the Synerigi Hydro-RP column (Phenomenex Inc., Torrance, CA, USA); methanol-water-acetic acid, 3:2:0.005 (v/v) as a solvent; a flow rate of 0.40 mL/min; an injection volume of 5.0 µL; the API2000 mass spectrometer (AB Sciex Pte. Ltd., Tokyo, Japan); electrospray ionization (ESI), in the positive mode; a spray pressure of 5,500 V; a turbo heater temperature of 400°C; and a selected ion of *m/z* 226.2→72.2, 229.2→75.2 (internal standard). Quantitative analysis via multiple reaction monitoring was performed using the MultiQuant software (AB Sciex Pte. Ltd.). The average of three LC-MS/MS measurements was used as the value of a given sample.

### 2.6 Recovery Test

For the 3-MBA derivatization method, the acrylamide standard was added to 5.0 g of fresh mung bean sprouts to a concentration of 145 ng/g for the recovery test, and the test was repeated 16 times.

For the thiosalicyclic acid derivatization method, the acrylamide standard was added to 5.0 g of fresh mung bean sprouts to a concentration of 50 ng/g for the recovery test, and the test was repeated seven times. Fresh mung bean samples without the added acrylamide standard and the acrylamide standard alone without mung bean sprouts were also analyzed to compensate for the effect of the small amounts of inhibitor contained in the sprouts on the acrylamide quantification results.

The limit of detection (LOD) and limit of quantification (LOQ) were calculated using the following equations:

LOD = *s* ×
*t***_(n−1, 0.05)_** ×
2

LOQ = *s* × 10

where *s* is the standard deviation of the analytical value, *t*_(n−1, 0.05)_ is the *t* value at the degree of freedom n−1 for a significance level of 0.05, and n is the number of test repetitions.

## 3. Results

### 3.1 Recovery Test

The recovery obtained using the 3-MBA derivatization method was 98%–103%. The LOD and LOQ were 29 and 74 ng/g, respectively.

The recovery obtained using the thiosalicyclic acid derivatization method was 112%–139%. The LOD and LOQ were 16 and 42 ng/g, respectively.

### 3.2 Acrylamide Formation in Mung Bean Sprouts during Stir-frying

**Fig. 1.**
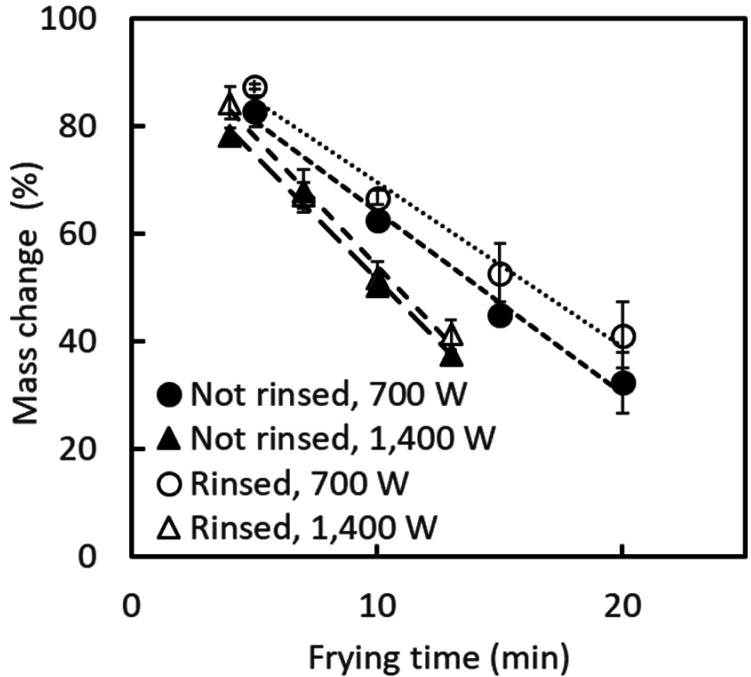
Mass change rate of mung bean sprouts during stir-frying. Error bars indicate standard deviation (n = 3).

The mass of mung bean sprouts decreased linearly with the heating time for all conditions (**Fig. 1**). The decreasing rate was greater when the IH heater was operated at 1,400 W than when it was operated at 700 W.

**Fig. 2.**
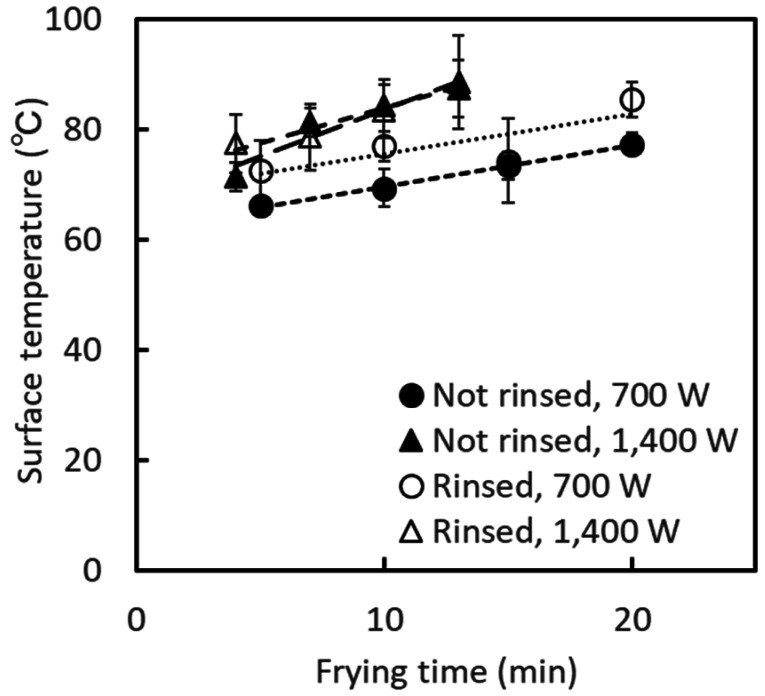
Change in the surface temperature of mung bean sprouts during stir-frying. Error bars indicate standard deviation (n = 3).

**Fig. 3.**
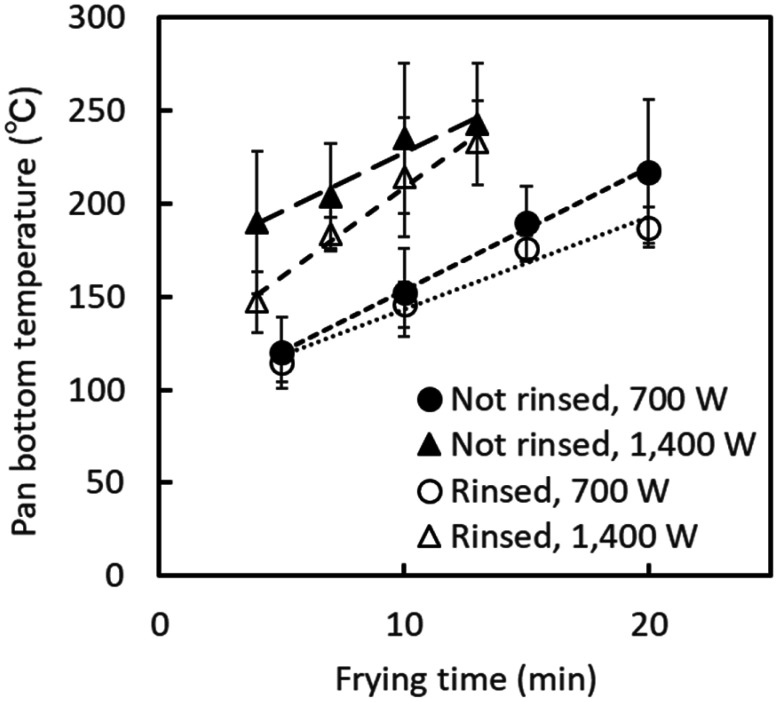
Change in the pan bottom temperature during stir-frying. Error bars indicate standard deviation (n = 3).

Although the surface temperature of the mung bean sprouts increased with the heating time, the average temperature remained low (below 100°C) for all conditions (Fig. 2). The surface temperature of the sprouts when the IH heater was operated at 700 W was lower than that at 1,400 W at that temperature. The temperature at the bottom of the pan increased linearly with the heating time (Fig. 3). It reached over 140°C and over 200 °C, on average, by operating the IH heater at 700 W for 10 min and at 1,400 W for 10 min, respectively. The bottom temperature was higher when the heater was operated at 1,400 than when it was operated at 700 W. Rinsing the sprouts before frying retarded the increase in the bottom temperature.

**Fig. 4.**
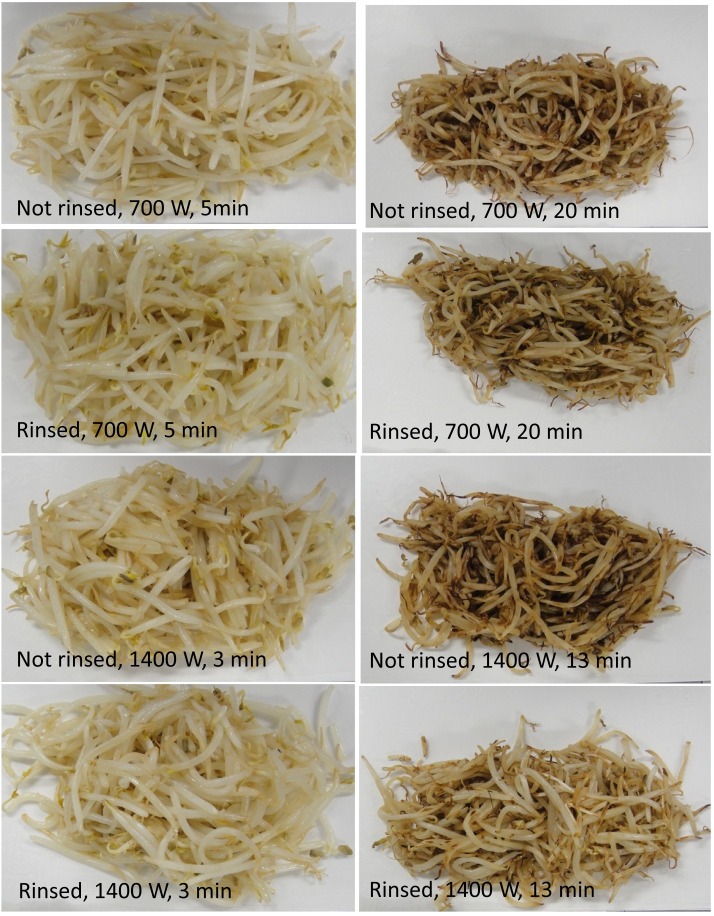
Photos of the stir-fried mung bean sprout samples.

Fig. 4 shows photographs of the stir-fried mung bean sprout samples. Shriveling due to moisture loss and browning due to the Maillard reaction were observed during the heating process, and all samples were considered edible.

**Fig. 5.**
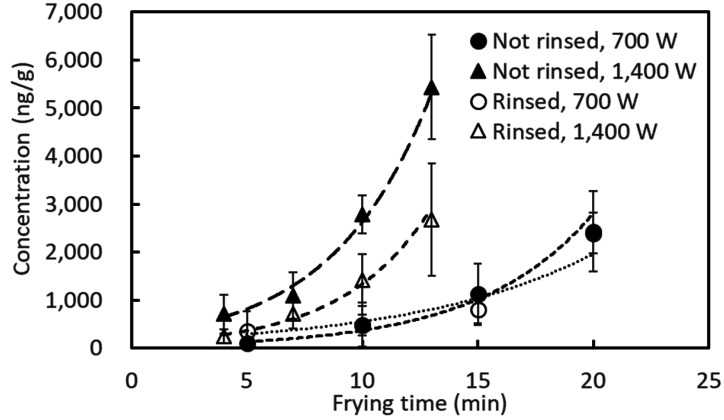
Acrylamide concentration of the stir-fried mung bean sprouts. When the acrylamide concentration was between the LOD and LOQ, its value was considered as the average of the LOD and LOQ. When the acrylamide concentration was below the LOD, it was considered as half of the LOD. Error bars indicate standard deviation (n = 3).

The acrylamide concentration in the sprouts increased exponentially with the heating time (Fig. 5). The heating of the mung bean sprouts that had not been rinsed at 1,400 W for 13 min generated an acrylamide concentration of over 5,000 ng/g, on average, whereas the rinsing of the sprouts before frying decreased this value by more than a half. When operating the IH heater at 700 W, the average acrylamide concentration exceeded 2,000 ng/g after 20 min of frying, regardless of having rinsed the sprouts before heating or not. Conversely, by operating the IH heater at 700 W and heating the sprouts for 15 min, their average acrylamide content remained approximately 1,000 ng/g. Frying the sprouts within 5 min limited the formation of acrylamide to below 1,000 ng/g, except for one replicate of non-rinsed sprouts fried by operating the IH heater at 1,400 W. The acrylamide content was below the LOQ (74 ng/g) for some cases in which the sprouts were heated within 5 min.

### 3.3 Acrylamide Concentration in Mung Bean Sprouts Cooked by Retaining Their Fresh Firm Texture

We investigated the levels of acrylamide formation by four cooking methods retaining the fresh firm texture using rinsed mung bean sprouts. The average mass change rate after cooking was 80.9% and 80.5% by stir-frying and parching, respectively, which was lower than the moisture loss induced by boiling (88.7%) or by cooking using a microwave oven (89.7%)(**Table 1**). The fried sprouts whose fresh firm texture was retained were slightly brownish; however, these did not shrivel. The appearance of the oven-cooked sample was similar to that of the stir-fried sample. The color of the parched mung bean sprouts was slightly darker; however, they did not shrivel. The boiled mung bean sprouts were white and did not shrivel.

**Table 1.** Mass change and Acrylamide Concentration of the Mung Bean Sprouts Cooked by Retaining their Fresh Firm Texture.

	Mass change rate (%)*	Acrylamide (ng/g)
Stir-fry (700 W, 5 min)	80.9 ± 0.5	< 42**
Parch (700 W, 5 min)	80.5 ± 0.5	< 42
Boil (1.5 min)	88.7 ± 0.2	< 42
Microwave oven cooking (500 W, 2 min)	89.7 ± 0.1	< 16

* The values are expressed as mean ± standard deviation (n=3).

**The analytical value of one of the three replicates was 42 ng/g.

The acrylamide concentration in microwave-oven-cooked sprouts was below the LOD. The other samples contained acrylamide at a concentration above the LOD but below the LOQ, except for one stir-fried sample replicate, whose acrylamide concentration was 42 ng/g.

## Discussion

The mass of mung bean sprouts decreased linearly (**Fig. 1**), which was also reported by Noda et al^13^^)^, and their color became brown (**Fig. 4**) with the stir-frying time. The surface temperature of the sprouts did not reach 100°C, even when the heater was operated at 1,400 W, except for one replicate sample heated for 13 min, because of the presence of moisture in the sprouts (**Fig. 2**). Conversely, the pan bottom reached a temperature above 140°C and above 200°C, on average, after frying the mung bean sprouts at 700 W for 13 min, and at 1,400 W for 10 min, respectively (**Fig. 3**). These high frying pan surface temperatures accelerated the formation of acrylamide in the fried food. Rinsing the sprouts before heating them at 1,400 W halved the amount of acrylamide formed (**Fig. 5**). Acrylamide mitigation by rinsing the vegetables prior to cooking them at a high temperature is described in a booklet for home cooking distributed by MAFF^15^^)^. The effectiveness of rinsing the sprouts with water to mitigate acrylamide formation was confirmed in this study. Rinsing increases the moisture content and decreases the asparagine and reducing sugar contents of the surface of the sprouts, which are key compounds for acrylamide formation^15^^)^.

Stir-frying mung bean sprouts using high heat (1,400 W) for 13 min without rinsing them led to the formation of 5,431 ng/g of acrylamide, on average, whereas stir-frying them after rinsing on medium heat (700 W) led to the formation of 807 ng/g of acrylamide, even after 15 min of cooking (Fig. 5). However, stir-frying the mung bean sprouts for longer than 10 min shriveled them, and their fresh texture was lost.

Since the triplicate time-course stir-frying experiment in this study was performed by three students, the variation in the results obtained also reflects the person-to-person variability in the cooking technique used, especially the mixing method. The variation in acrylamide content was especially large when a large amount of acrylamide was formed by long-term heating. For example, a standard deviation of more than 1,000 ng/g was observed after heating the sprouts at 1,400 W for 13 min (Fig. 5). A standard deviation of more than 800 ng/g was also observed, even after heating the sprouts using medium heat (700 W) for a long period, such as 20 min.

By short-time stir-frying (within 5 min) the sprouts, the formation of acrylamide sometimes did not reach 74 ng/g, the LOQ of the 3-MBA derivatization method. However, the corresponding standard deviation was 108–421 ng/g. This large variation is due to the variation in the asparagine and sugar contents of the sprouts, as well as from the mixing method used. When the mixing was inhomogeneous, and the sprouts were burned, a high acrylamide concentration was formed in the burned parts. The relative standard deviation, which is calculated by dividing the standard deviation by the mean, was in the 57%–121% range for the sprouts cooked within 5 min. This is larger than that obtained for the sprouts cooked for 13 min or more (18%–54%), which shows that the variation in the acrylamide amount formed is also large under short-time frying conditions.

The acrylamide concentration in mung bean sprouts cooked for a short time while retaining their fresh firm texture was less than 42 ng/g, on average. Therefore, the minimum value of 28 ng/g reported in the Survey Results of Hazardous Chemicals in Foods (2011-2012) by MAFF^10^^)^ and the 1–35 µg/kg (ng/g) concentration range reported in the total diet study done in Hong Kong^6^^)^ are regarded as values that are generally observed in stir-fried mung bean sprouts. Noda et al^13^^)^ parched mung bean sprouts and reported that their acrylamide content was 1,250 ng/g after heating them for 3 min. This is considered to be the value found in shriveled sprouts cooked under high heat. They parched 100 g of sprouts using a frying pan with a diameter of 24 cm, whereas we parched 200 g of sprouts using a pan with a bottom diameter of 20 cm. The thermal conduction to sprouts may have been more effective in the former experiment. A wide range of acrylamide concentration values (from < 74 (LOQ) to 1,270 ng/g) was observed after heating the sprouts at 1,400 W for 4 min in our experiment (Fig. 5). These results indicate that the acrylamide concentration varies significantly with the changes in color and texture, even for a short-time frying, depending on the cooking condition.

In the study by Noda et al^13^^)^, five students perched mung bean sprouts as if they would eat them, and the resulting acrylamide concentration range of the sprouts was 2.7–92 µg/100 g (27–920 ng/g) raw sprout basis. This variation suggests that some of the five students perched the mung bean sprouts quickly at low or medium heat, avoiding deteriorating the fresh texture of the sprouts. Ono^11^^)^ asked 20 people to stir fry mung bean sprouts twice as they liked and analyzed the acrylamide content of 40 samples, resulting in a minimum, maximum, median, and average concentration of 22, 570, 115, and 135 ng/g, respectively, and a standard deviation of 110 ng/g. The acrylamide concentration in 20% (8/40) and 72% (29/40) of the samples was less than 50 ng/g and less than 150 ng/g, respectively, and exceeded 500 ng/g in only one sample.

Considering that a large percentage of Japanese people usually stir-fry mung bean sprouts quickly to retain their fresh texture, the oral intake of acrylamide, 0.240 µg/kg bw/day, which is revised point estimation by FSCJ calculated using the acrylamide content of 752 ng/g in mung bean sprouts (fried/sautéed)^8^^,^^9^^)^, should be an overestimation. The earlier oral intake estimation of 0.154–158 µg/kg bw/day calculated using an acrylamide content of 95 ng/g in fried bean sprouts^8^^,^^9^^)^ seems more realistic. In the former case, bean sprouts (fried/sautéed) were the food that contributed the most to the total acrylamide intake, constituting 28% (66 ng/kg bw/day) of the total intake. The contribution of the bean sprouts (fried/sautéed) became only 5% (8.4–8.5 ng/kg bw/day) in the latter case, which is lower than those of coffee and potatoes (fried), showing that mung bean sprouts are a lower priority in acrylamide risk management. The value 42 ng/g, the maximum acrylamide concentration of the bean sprouts stir-fried while retaining their fresh texture in this study, was replaced with 752 ng/g, the value used for revised exposure estimation by FSCJ^8^^,^^9^^)^. The calculated total acrylamide intake decreased from 0.240 to 0.178 µg/kg bw/day, for which the contribution of bean sprouts was 2% (3.7 ng/kg bw/day). This contribution was 17th from the highest and lower than those of wheat flour snacks, potato chips, rice crackers, green tea/oolong tea, and cooked rice. Thus, the representative value of fried bean sprouts used for intake estimation strongly affects their importance and priority in risk management of acrylamide in food. The total acrylamide intake in Japan remains around 0.2 µg/kg bw/day in all the above cases, and the margin of exposure for neoplastic effects ranges from 700 to 2,000. Thus, the result of the risk assessment by FSCJ, “continual efforts are necessary to reduce dietary acrylamide intake in accordance with the principle of ALARA (as low as reasonably achievable) from the viewpoint of public health” is still valid.

Adopting a certain cooking condition forming or containing the target hazard most is reasonable to avoid underestimating its intake in risk assessment. However, knowing the ranges of the parameters of popular cooking methods and the resulting realistic range of the concentration of the target hazard based on a wide survey for each food item is necessary to identify an appropriate representative concentration value or the concentration distribution in each item for accurate risk characterization. Risk assessment based on an accurate risk characterization is essential for effective risk management. The acrylamide concentration range in stir-fried mung bean sprouts is especially broad, even within the range of general cooking conditions, and thus, selecting an adequate representative concentration value is difficult. A more precise survey and the accumulation of data on acrylamide formation in relation to the components present before heating, their changes during storage, and the cooking conditions are required for the risk assessment on acrylamide in bean sprouts.

When mung bean sprouts are stir-fried with meat and other vegetables under high heat for a long time, the resulting acrylamide formed is considered from this study to be 100 times that formed in sprouts fried for a short period that retain their fresh texture. Based on the ALARA principle, mung bean sprouts should be put in the pan separately at the timing when the meat and other vegetables are almost cooked. Removing the sugars and amino acids on the surface of vegetables by rinsing them before frying is also an effective way to mitigate acrylamide formation, as suggested in a booklet by MAFF^15^^)^. Mixing well during frying to avoid burning any parts of the food through long-time contact of the food material with the surface of the pan is important for further inhibiting acrylamide formation. To decrease the microbial risk, the bean sprouts should be heated before serving, even when used in salads, by short-time perching, boiling, or microwave-oven heating them.

## References

[1] Tareke E, Rydberg P, Karlsson P, Eriksson S, Törnqvist M. Analysis of acrylamide, a carcinogen formed in heated foodstuffs. J Agric Food Chem. 2002; 50(17): 4998–5006. . 10.1021/jf020302f 12166997

[2] Ahn JS, Castle L, Clarke DB, Lloyd AS, Philo MR, Speck DR. Verification of the findings of acrylamide in heated foods. Food Addit Contam. 2002; 19(12): 1116–1124. . 10.1080/0265203021000048214 12623671

[3] European Food Safety Authority (EFSA) Panel on Contaminants in the Food Chain. Scientific Opinion on acrylamide in food. EFSA J. 2015; 13(6): 4104. . 10.2903/j.efsa.2015.4104

[4] Abt E, Robin LP, McGrath S, et al. Acrylamide levels and dietary exposure from foods in the United States, an update based on 2011-2015 data. Food Addit Contam Part A Chem Anal Control Expo Risk Assess. 2019; 36(10): 1475–1490. . 10.1080/19440049.2019.1637548 31318642

[5] Food Standards Australia New Zealand. 24th Australian Total Diet Study. Part D – Acrylamide. 2014; 31-42. https://www.foodstandards.gov.au/publications/Documents/1778-FSANZ_AustDietStudy-web.pdf. Accessed on January 21, 2023

[6] Centre for Food Safety, Food and Environmental Hygiene Department, The Government of the Hong Kong Special Administrative Region. The First Hong Kong Total Diet Study: Acrylamide, The First Hong Kong Total Diet Study Report No. 6. 2013. https://www.cfs.gov.hk/english/programme/programme_firm/files/The_first_HKTDS_acrylamide_final_e.pdf. Accessed on January 21, 2023

[7] Zhu F, Wang Y, Liu H, et al. Exposure to Acrylamide in the Sixth Total Diet Study — China, 2016–2019. China CDC Wkly. 2022; 4(9): 161–164. . 10.46234/ccdcw2022.040 35356409 PMC8930407

[8] Food Safety Commission of Japan. Risk Assessment Report. Acrylamide in foods generated through heating [in Japanese]. 2016. http://www.fsc.go.jp/fsciis/attachedFile/download?retrievalId=kya20160405231&fileId=200. Accessed on January 21, 2023

[9] Food Safety Commission of Japan. Acrylamide in foods generated through heating. Food Safety. 2016; 4(3): 74–88. . 10.14252/foodsafetyfscj.2016013s 32231909 PMC6989166

[10] Ministry of Agriculture, Forestry and Fisheries. Survey results of hazardous chemicals in foods (2011-2012) [in Japanese]. 2014. https://www.maff.go.jp/j/syouan/seisaku/risk_analysis/survei/pdf/chem_23-24_.pdf. Accessed on January 21, 2023

[11] Ono H. Report on the cooking methods to reduce acrylamide in heating. Regulatory science research project [in Japanese]. 2015. https://www.maff.go.jp/j/syouan/seisaku/regulatory_science/shuryo_chem.html#aa. Accessed on January 21, 2023

[12] Ishihara K, Nara K, Yonezawa Y, Hoshino A, Arima M, Koga H. Acrylamide content in vegetables home-cooked by heating [in Japanese]. Journal of Cookery Science of Japan. 2009; 42(1): 32–37. https://dl.ndl.go.jp/view/download/digidepo_10815497_po_ART0009052030.pdf?contentNo=1&alternativeNo=.

[13] Noda K, Ando H, Tada K, et al. Acrylamide formation during pan-frying of mung bean sprouts. Food Sci Technol Res. 2022; 28(4): 307–315. . 10.3136/fstr.FSTR-D-21-00030

[14] Jezussek M, Schieberle P. A new LC/MS-method for the quantitation of acrylamide based on a stable isotope dilution assay and derivatization with 2-mercaptobenzoic acid. Comparison with two GC/MS methods. J Agric Food Chem. 2003; 51(27): 7866–7871. . 10.1021/jf0349228 14690366

[15] Ministry of Agriculture, Forestry and Fisheries (MAFF). For safe and healthy eating. What we can do at home to reduce acrylamide in foods. 2015. https://www.maff.go.jp/j/syouan/seisaku/acryl_amide/a_syosai/teigen/pdf/aa_booklet_en.pdf.

